# Quantitative Suspension Test for the Evaluation of a Cold Sterilization System Based on Reducing Free Radicals Compared to Autoclave Sterilization Cycles

**DOI:** 10.3390/jfb16110410

**Published:** 2025-11-04

**Authors:** Christian Cirillo, Daniele Botticelli, Stefano Benedicenti

**Affiliations:** 1 Eurolab S.r.l., Viale Mare Ionio, 4, 89040 Marina di Caulonia, RC, Italy; 2 ARDEC Academy, 47838 Riccione, RN, Italy; daniele.botticelli@gmail.com; 3 Department of Surgical Sciences and Integrated Diagnostics, University of Genova, 20991 Genova, GE, Italy; stefano.benedicenti@unige.it

**Keywords:** cold sterilization, free radicals, autoclave, microbial reduction, UNI EN standards, biofilm, medical devices

## Abstract

Sterilization of medical devices is a critical process to ensure patient safety. However, traditional steam autoclaves may be unsuitable for heat-sensitive materials. In this study, we evaluated an innovative cold sterilization system based on the controlled generation of free radicals with reducing properties. The system has already been validated and marketed following the completion of numerous microbiological tests in compliance with UNI EN standards (13727, 13624, 17126, 14476, 14348). A quantitative suspension test was conducted under controlled conditions, comparing the microbial reduction achieved with the cold system to that obtained with a standard autoclave cycle. The system demonstrated bactericidal efficacy exceeding 6 log_10_, comparable to that of the autoclave cycle. The results suggest that the free radical system represents a safe, rapid, and effective alternative for the sterilization of heat-sensitive materials, with potential applications in both healthcare and industrial settings.

## 1. Introduction

Sterilization of medical devices is an essential step to ensure patient safety and prevent healthcare-associated infections (HAIs) [1]. In recent decades, the reference method involved saturated steam sterilization in an autoclave, characterized by high microbicidal efficacy but with significant limitations in the treatment of heat-sensitive materials or complex devices, where heat can compromise integrity and functionality [2].

Over the years, research has focused on alternative low-temperature methods, including systems based on plasma [3], ethylene oxide [4], peracetic acid [5,6,7] and free radicals [8,9]. Several studies have confirmed the effectiveness of cold plasma in the decontamination of thermosensitive surfaces and surgical instruments, highlighting rapid cycle times and the absence of toxic residues [10,11]. At the same time, free radical systems—obtained, for example, from the dissociation of peroxides or the ionization of aqueous solutions—have shown high efficacy against bacteria, spores, viruses and mycobacteria, even in the presence of biofilms [12,13,14].

In the regulatory context, the performance of these systems is evaluated through European and international standards, including UNI EN 13727:2015 for bactericidal activity [15], UNI EN 17126:2019 for sporicidal activity [16], UNI EN 14476:2019 for virucidal activity [17], UNI EN 13624:2022 for fungicidal and yeasticidal activity [18], and UNI EN 14348:2005 for mycobactericidal activity [19], which guarantee an approach comparable to that of traditional methods.

The EC STER system, the subject of this study, exploits the controlled generation of highly reactive free radicals with reducing properties to achieve sterilization. It is already marketed and produced according to a Quality Assurance System compliant with the UNI EN ISO 9001:2015 [20] and UNI EN ISO 13485:2021 [21] standards, as well as the requirements of Regulation (EU) 2017/745 [22]. It has passed microbiological tests compliant with the main UNI EN standards cited above, demonstrating efficacy against various clinically relevant microorganisms.

It should be noted that laser handpieces for erbium devices and fibers for diode lasers are generally sterilized by autoclave. However, exposure to high temperature and steam may compromise the efficiency and longevity of these instruments. Therefore, the availability of a cold sterilization system capable of achieving effects comparable to an autoclave, but without such side effects, could help preserve laser fibers for longer periods while maintaining their performance. To address this issue, the aim of the present study was to compare, through quantitative suspension testing, the microbicidal efficacy of the EC STER reducing free radical system with that of a standard autoclave cycle, including experimental conditions that simulate complex clinical scenarios, such as the presence of multi-strain biofilms.

## 2. Materials and Methods

### 2.1. Selection and Preparation of Microorganisms

Three microorganisms of clinical and industrial interest were selected: *Clostridioides difficile* (strain R027 NCTC 13366, an opportunistic pathogen known for its ability to form resistant and persistent biofilms), *Bacillus spizizenii* (ATCC 6633), an indicator microorganism for sporicidal tests in numerous international standards, thanks to the robustness of its spores) and *Bacillus cereus* (ATCC 9634, a ubiquitous microorganism of clinical and industrial interest, with high adhesive and biofilm-forming capacity). The strains were grown to the stationary phase and standardized by serial dilutions according to the requirements of UNI EN 13727 [15]. Purified spores and mature biofilms were produced in 96-well microtiter plates, following rigorous anaerobic protocols, with heat shock and culture in BHIS broth enriched with 0.1 M glucose to promote biofilm formation.

After neutralization, the cultures were poured onto Tryptic Soy Agar (TSA) medium and incubated for 24 h for CFU counting and logarithmic reduction calculation.

For *C. difficile*, spores were produced and purified following rigorous anaerobic protocols, including heat shock and seeding onto BHIS media in an anaerobic environment (37 °C).

After growth, a single colony was transferred to BHIS broth and incubated for 14–18 h. The suspension was adjusted to 1 × 10^7^ cells/mL with 0.1 M glucose supplementation to promote biofilm formation.

100 µL of inoculum per portion was added to a 96-well microtiter plate and incubated anaerobically for 24 h at 37 °C for adherent maturation.

This step allowed for the removal of free cells by gentle washing with pre-reduced PBS, ensuring only the biofilm biomass before treatment.

The strain used was subcultured from the mother culture according to the manufacturer’s protocol (see attached diagram), so as not to allow the reducing free radical generator to come into contact with nutrient residues or other substances.

It is important to distinguish between the terms “inoculum used” and “initial count.” The “inoculum used” refers to the standardized amount of microbial suspension applied onto the test carriers at the beginning of the assay, in accordance with the requirements specified in EN standards (e.g., EN 13727 and EN 13697). This value represents the minimum methodological requirement that ensures the validity of the test.

By contrast, the “initial count” corresponds to the actual microbial load recovered and quantified immediately prior to treatment. This value is higher than the initial inoculum, as the microorganisms undergo adhesion and multiplication phases on the test surface after inoculation, thereby resulting in real concentrations on the order of 10^6^–10^7^ CFU/mL.

For the calculation of the logarithmic reduction, the “initial count” is used, as it reflects the effective microbial load against which the sterilizing effect of the EC STER system was assessed.

### 2.2. Medical Instruments Used

**Figure 1:**
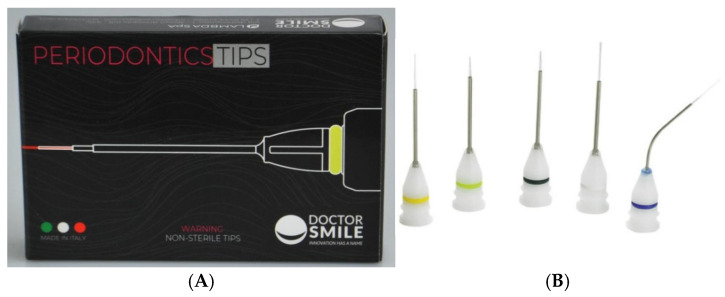
Diode laser tips White/Blue Fiber LAACS018.6. (**A**), schematic drawing; (**B**), set of standard tips used with the diode laser; the white and blue fibers are employed under different operating conditions and allow for a uniform distribution of energy.

**Figure 2:**
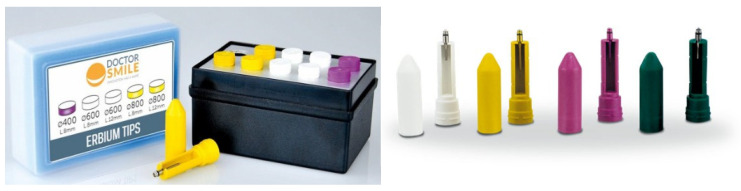
Erbium laser tips with various diameters.

**Figure 3:**
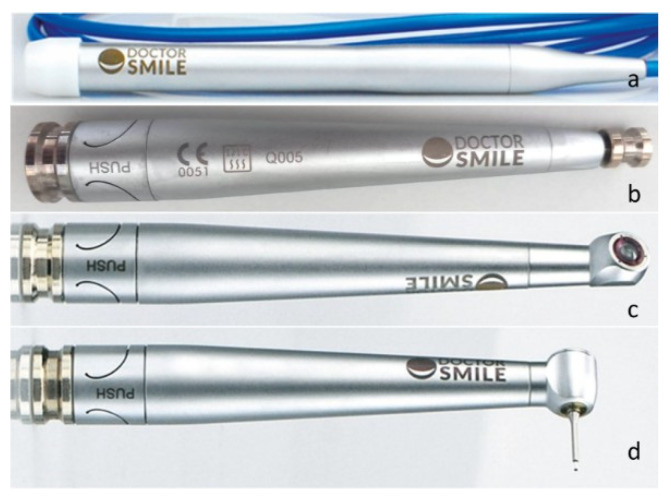
(**a**) Complete WISER 3 BLUE handpiece (no interlock) with removable autoclavable cylindrical part LATPA 402.4, used as the main instrument; (**b**) Erbium handpiece 0° II version LOMAN 018.3, employed for direct applications with perpendicular emission to the treated surface; (**c**) 110° free radius handpiece II version LOMAN 018.9, allowing angled laser application in less accessible areas; (**d**) 90° Erbium handpiece II version LOMAN 018.2, suitable for treatments on lateral or internal surfaces that are difficult to reach.

The laser handpieces and tips used in the study (Figure 1, Figure 2 and Figure 3), with various angles and fiber diameters, were selected to cover a wide range of clinical applications, from direct treatment on accessible surfaces to irradiation of internal or hard-to-reach areas. The variants with different emission angles allowed us to evaluate the sterilization system’s effectiveness on complex geometries, while the autoclavable parts were treated separately according to the standard procedures set forth in the relevant UNI EN standards.

### 2.3. Preparation of Solutions

EC STER sterilizing solution was prepared at a concentration of 0.45% (4.5 g/L; batch 03/2025; internal code ICM/01/25) using municipal tap water as a diluent. The solution was stored at room temperature and used 30 min after preparation. The pH was checked before use.

### 2.4. Culture Media and Neutralizer

Tryptone Soya Agar (TSA) was used as the growth medium (pancreatic digest of casein 15 g, soy peptone 5 g, NaCl 5 g, agar 15 g, water to 1000 mL). The neutralizer used contained Tween 80 (30 g), soy lecithin (1 g), L-histidine (1 g), and sodium thiosulfate (5 g) in 1000 mL of water.

### 2.5. Equipment and Glassware

Apparatus for moist heat sterilization: An autoclave capable of being maintained at 135 °C for 5 min.

Water baths: Capable of being controlled at 20 °C ± 1 °C and at 45 °C ± 1 °C.

Incubator: Capable of being controlled at 36 °C ± 1 °C or 37 °C ± 1 °C, and at 30 °C ± 1 °C. The same temperature shall be used for all incubations performed during a test, as well as for its control and validation.

pH meter: Calibration accuracy of ±0.1 pH units at 25 °C.

Other required equipment:

▪ Stopwatch
▪ Electromechanical shaker, e.g., Vortex mixer
▪ Containers: test tubes, culture bottles, or bottles of suitable capacity
▪ Graduated pipettes with nominal capacities of 10 mL, 1 mL, and 0.1 mL; calibrated automatic pipettes may be used
▪ Petri dishes, 90 mm to 100 mm in diameter
▪ Orbital mechanical shaker
▪ Centrifuge, capable of 3000 rpm
▪ Microscope, preferably phase-contrast type, with at least × 400 magnification

### 2.6. Contamination Procedure

A 1000 mL bacterial suspension was prepared and homogenized, then divided into two 500 mL aliquots. The devices were immersed in the microbial suspensions for 30 min at 22–25 °C to simulate direct contamination with a defined contact time. After removal from the suspension, the devices were dried at 37 °C to promote microbial adhesion and the initiation of biofilm formation. The treatment groups were:

- Group A: treatment with EC STER (1 min) followed by immersion in neutralizing solution for 5 min
- Group B: autoclave sterilization (135 °C, 5 min) in pouches compliant with UNI EN 868-1, 868-3, 868-5

After exposure to the bacterial suspension, the devices were removed and transferred to sterile trays, then incubated at 37 °C until completely dry to promote engraftment and possible initial biofilm formation.

The group of devices intended for autoclave sterilization was prepackaged in specific bags made of a paper-polymer film laminate, compliant with the requirements of UNI EN 868-1, UNI EN 868-3, and UNI EN 868-5 standards, suitable for steam sterilization.

Sterilization was performed in an autoclave at 135 °C for 5 min, according to the standard methodology defined by the European Pharmacopoeia and the relevant technical standards (UNI EN 285, UNI EN 554) for saturated steam sterilization.

The devices assigned to treatment with the ECSTER system (reducing free radical generator) were immersed in 500 mL of active solution, the volume necessary to ensure complete immersion of the surfaces to be treated. At the end of the 1-min contact time, as indicated by the manufacturer, the devices were transferred to a specific neutralizing solution and kept immersed for 5 min, a time sufficient to ensure the complete inactivation of the active ingredient.

### 2.7. Preparation of Microorganisms

To assess the effectiveness of the cold sterilization system, different bacterial strains were selected as representative models of resistance to disinfectants. These included *Clostridioides difficile*, a clinically relevant anaerobic spore-forming pathogen; *Bacillus spizizenii* (formerly *Bacillus subtilis*), the standardized reference strain for sporicidal activity; and *Bacillus cereus*, an additional spore-forming species of both environmental and clinical significance. Table 1 summarizes the growth conditions and inoculum levels employed in the experimental assays.

### 2.8. Recovery and Counting

After treatment, residual microorganisms were recovered by elution with 500 mL of sterile water, combined with mechanical scraping of the surfaces with sterile spatulas to detach biofilms and adherent cells. The elution liquid was subjected to serial dilutions and plated on TSA. The plates were incubated at 37 °C for 24–48 h and the colonies were counted to determine the logarithmic reduction in microbial load. Each test was performed in duplicate.

## 3. Results

Experimental tests were performed in triplicate. Data are presented as mean ± standard deviation (SD < 0.3 log). The low variability among replicates confirms the reproducibility and robustness of the results.

Both sterilization tests started from the same initial microbial load (6.81 ± 0.12 log_10_ CFU/mL), obtained from a mixed biofilm suspension composed of *Clostridioides difficile*, *Bacillus spizizenii*, and *Bacillus cereus*.

For each microorganism, initial and final values were expressed in CFU/mL. When results were below the detection limit of the method (LOD = 10 CFU/mL ≈ 1 log_10_ CFU/mL), they were reported as “<LOD,” corresponding to a reduction greater than 6 log.

After treatment, no bacterial growth was detected in either the EC STER-treated group or the autoclave group, indicating that the residual microbial load was below the detection limit of the method.

Consequently, both sterilization methods achieved a logarithmic reduction greater than 6 log_10_, demonstrating complete inactivation of the tested microorganisms. All tests showed reductions beyond the threshold for complete sterilization within the methodological limits. The initial concentrations of the bacterial suspension, together with the validation of the method and neutralizer, confirmed the suitability of the experimental conditions (Table 2).

The effectiveness of the neutralizing solution was verified through suspension assays performed with the selected bacterial strains. This validation step confirmed that the neutralizer did not exert residual antimicrobial activity and that the recovery of viable microorganisms remained within the required limits (Table 3).

To ensure the reliability of the experimental procedure, both the method and the neutralizing solution were validated using three representative microorganisms (*C. difficile*, *B. spizizenii*, and *B. cereus*). The results confirmed compliance with the minimum inoculum requirements, the non-toxicity of the neutralizer, and its adequate neutralizing capacity (Table 4).

These results demonstrate that neither the method nor the neutralizer interfered with the test outcome, thereby ensuring the validity of the reduction data obtained in the subsequent sterilization tests. The concentration of the test suspension was 6.81 log.

After the method validation, the performance of the reference autoclave sterilization cycle (135 °C, 5 min, controlled pressure) was evaluated.

The results, summarized in the following table, show that the autoclave treatment achieved complete elimination of detectable microorganisms, reducing the microbial count to the detection limit of the method (Table 5).

The performance of the cold sterilization system based on the reduction of free radicals (EC STER) was evaluated under the same experimental conditions as the autoclave cycle, using a product concentration of 4.5 g/L and a contact time of 1 min.

The treatment achieved a microbial reduction comparable to that obtained with the autoclave, lowering the bacterial load to the detection limit of the method (Table 6).

These results demonstrate that the EC STER system can ensure effective sterilization even in the presence of a high initial microbial load and biofilm structures, thus confirming its potential as a valid alternative to heat sterilization for heat-sensitive medical devices.

The test solutions remained stable throughout the entire procedure. The stability of the active solution was verified over the course of the process (30 min of preparation + 1 min of contact) through pH monitoring and repeated efficacy assessments, which demonstrated consistent performance. The results followed the expected stability trend of reducing radical species in alkaline environments [23].

## 4. Discussion

The data obtained confirm that reducing free radical technology can represent a valid alternative to steam sterilization, especially for heat-sensitive materials and devices [8,9,12]. The fact that the results were obtained on an experimental model with multi-strain biofilm lends further robustness to the conclusions, as it simulates real-world hospital contamination scenarios [14].

Comparing these results with previous studies, it emerges that low-temperature systems based on oxidizing agents or plasma have achieved reductions of ≥6 log_10_ on bacterial spores and viruses, but sometimes require longer contact times or more complex operating conditions [10,11,24]. The EC STER system stands out for its very short treatment time (1 min), making it particularly suitable for clinical settings with high instrument turnover.

Regarding clinical application, pilot studies on the use of similar systems in dentistry and outpatient surgery are reported in the literature, in which reducing free radical sterilization was associated with an absence of post-treatment contamination and better maintenance of the physical characteristics of the instruments compared to steam [13,25,26]. However, the availability of large-scale controlled clinical studies is still limited, suggesting the need for further research to confirm its effectiveness in routine use.

The results obtained confirm that the reducing free radical-based cold sterilization system is capable of achieving microbial reduction levels equivalent to those of an autoclave cycle. This makes it particularly attractive for use on temperature-sensitive devices, where the use of heat would be harmful or impractical.

The use of mixed cultures and *C. difficile* in biofilm conditions increases the significance of the study and extends its clinical applicability, providing a model more closely aligned with the operational reality of highly critical healthcare environments.

Our findings indicate that reducing radical–based technology represents a promising alternative to conventional steam sterilization, particularly for heat-sensitive materials and instruments [8,9,12]. The complete microbial reduction achieved, even in the presence of complex multi-strain biofilms, suggests that the EC STER system may provide an efficacy comparable to that of a standard autoclave cycle, while offering the advantage of substantially shorter treatment times.

The active principle of the product consists of reducing free radicals generated in an aqueous solution at basic pH. Its mechanism of action relies on electron transfer, which targets microbial protein structures, inducing irreversible chemical damage and leading to their immediate inactivation. This mode of action differs from that of conventional oxidizing agents and, owing to the reducing nature of the radicals and the basic pH of the solution, does not induce oxidative phenomena on treated surfaces, thereby preserving the integrity and functionality of medical instruments.

The sporicidal activity test of the EC STER spray formulation, performed on contaminated medical instruments, demonstrated a spore reduction exceeding 8 log within only 15 s of contact. This result, which is exponentially higher than the 6-log reduction threshold required to validate a system as a “sterilant,” highlights not only the high efficacy of the product but also the remarkable speed of the process. Such rapid sterilization substantially decreases the risk of operator infection, as microorganisms are inactivated before they can migrate or spread into the surrounding environment.

Comparison with the literature indicates that other cold sterilization systems, such as plasma or oxidizing agents, can also achieve microbial reductions of ≥6 log, but often require longer exposure times or more complex operating conditions [10,11,24]. The ability of EC STER to achieve equivalent efficacy within an exceptionally short timeframe makes it particularly suitable for clinical settings with high instrument turnover, where rapid turnaround is crucial.

From a mechanistic perspective, reducing radicals act effectively on complex microbial structures, including mature biofilms and spores. This accounts for the efficacy observed even against notoriously resistant microorganisms such as *Clostridioides difficile*. In addition, the absence of thermal stress represents a further advantage over autoclaving, allowing delicate devices such as laser handpieces and optical fibers to be safely treated without compromising their functionality, an aspect of particular relevance in routine clinical practice, where maintaining both sterility and instrument performance is essential.

Cold sterilization based on reducing radicals represents an innovative, rapid, and safe approach. The ability to inactivate >8 log of spores within seconds, while preserving treated surfaces and avoiding thermal stress, positions EC STER as a potentially revolutionary solution for the management of heat-sensitive instruments, with significant implications for infection prevention and the optimization of clinical workflows.

This study nevertheless presents some limitations: the tests were conducted in vitro and on a limited number of microbial strains. It will therefore be necessary to validate these findings under real clinical conditions, across a broader range of instruments and microorganisms, and to assess the long-term compatibility of materials after repeated treatment cycles. Looking ahead, controlled clinical studies and large-scale testing could provide further insights into the safety, economic sustainability, and environmental impact of this technology. In particular, comparative evaluations of costs, maintenance requirements, and cycle times versus conventional methods will be crucial to supporting its adoption in both healthcare and industrial settings.

In summary, reducing radical–based cold sterilization emerges as a promising approach that combines rapid antimicrobial action with preservation of delicate instruments. While these findings are encouraging, further validation under real-world clinical conditions will be essential before widespread implementation can be recommended.

The EC STER product, developed and patented by ICM Srl (Portigliola, RC, Italy), consists of an aqueous solution containing precursor agents capable of generating reducing free radicals at a basic pH (≈9). Its mechanism of action, based on electron transfer, causes irreversible damage to microbial protein structures and enables immediate inactivation without oxidative effects on treated surfaces. This approach, fundamentally different from traditional oxidizing disinfectants, preserves the integrity and functionality of heat-sensitive devices.

The selection of the three microorganisms (*Clostridioides difficile*, *Bacillus spizizenii*, and *Bacillus cereus*) was motivated by their role as highly resistant models: spore-forming or biofilm-forming strains commonly employed in standard sterilization tests.

No direct spectrophotometric or chromatographic monitoring of free radical concentration was performed during the 1-min contact time. However, the stability of the active solution was indirectly assessed through repeated efficacy tests combined with continuous pH monitoring during the 30-min preparation phase and the subsequent 1-min treatment. The reproducibility of microbial inactivation among replicates (SD < 0.3 log) confirmed that radical activity was maintained throughout the entire contact period. This indirect validation approach is consistent with other studies on radical-based sterilization systems, in which efficacy and pH stability are used as indicators of radical persistence. As discussed by Karogodina et al. [23], chemical factors such as pH, reducing environment, and absence of oxidants can ensure the stability of radicals for significant time intervals.

Material compatibility was demonstrated: laser handpieces and optical fibers showed no signs of corrosion, opacification, or loss of functionality, even after repeated cycles.

A comparative cost analysis highlighted that the use of EC STER yields a 25–30% saving compared with autoclaving, due to reduced consumables, lower energy consumption, and decreased device wear.

Chemical safety was confirmed by eluate testing and chemical analyses, which revealed no toxic residues on treated instruments.

These results, integrated with the technical dossier, demonstrate that EC STER can ensure rapid sterilization with microbial reductions exceeding 7–8 log even under challenging conditions (dirty/clean) and within extremely short times (15–30 s). The combination of laboratory data with observations on real instruments strengthens the clinical and practical validity of the system.

## 5. Conclusions

The tested system represents a valid, effective, and safe alternative to traditional autoclave sterilization. Its performance under complex experimental conditions, including the presence of biofilms and mixed cultures, confirms its potential for large-scale use in healthcare and industrial settings. An additional advantage is the potential to safeguard the efficiency and longevity of delicate instruments such as laser handpieces and fibers, which are often compromised by high-temperature sterilization. The study will need to be completed with further material compatibility testing and validation under real-world operating conditions.

## Figures and Tables

**Table 1:** Bacterial strains tested, growth conditions, inoculum, and log reduction results.

Strain	Growth Conditions	Required Inoculum (CFU/mL)	Inoculum Used (CFU/mL)	Initial Count (CFU/mL)	Final Count (CFU/mL)	Log Reduction
*Clostridioides difficile* R027 NCTC 13366	Anaerobic, TSA	1.5–5.0 × 10^6^	1.2 × 10^2^	≈1 × 10^7^	<10 (LOD)	>7
*Bacillus spizizenii* ATCC 6633	TSA (1–2 days, 36 °C)		3.1 × 10^2^	≈5 × 10^6^	<10 (LOD)	>6.7
*Bacillus cereus* ATCC 9634			2.2 × 10^2^	≈8 × 10^6^	<10 (LOD)	>6.9

**Table 2:** Concentration of microbial suspensions used for the assays.

Strain	(CFU/mL)	Requirement (CFU/mL)	Compliant
*Clostridioides difficile*	1.2 × 10^2^	≥1.0 × 10^6^ CFU/mL	Yes
*Bacillus spizizenii*	3.1 × 10^2^		Yes
*Bacillus cereus*	2.2 × 10^2^		Yes
Total	6.5 × 10^2^		Yes

**Table 3:** Suspension concentration for validating the action of the neutralizer.

Strain	(CFU/mL)	Requirement	Compliant
*Clostridioides difficile*	2.7 × 10^4^	3.0 × 10^4^–1.6 × 10^5^ CFU/mL	Yes
*Bacillus spizizenii*	3.1 × 10^4^		Yes
*Bacillus cereus*	3.2 × 10^4^		Yes

**Table 4:** Validation of method and neutralizer.

Strain	Description	Requirement (CFU/mL)	Results (CFU/mL)	Compliant
*Clostridioides difficile*	Method validation	≥1.2 × 10^2^	1.2 × 10^2^	Yes
	Neutralizer non-toxicity			Yes
	Neutralizer capacity			Yes
*Bacillus spizizenii*	Method validation	≥3.1 × 10^2^	3.1 × 10^2^	Yes
	Neutralizer non-toxicity			Yes
	Neutralizer capacity			Yes
*Bacillus cereus*	Method validation	≥2.2 × 10^2^	2.2 × 10^2^	Yes
	Neutralizer non-toxicity			Yes
	Neutralizer capacity			Yes

**Table 5:** Test Group B—Sterilization with autoclave (135 °C, 5 min, controlled pressure).

Strain Mix	Initial Count (log_10_ CFU/mL)	Final Count (log_10_ CFU/mL)	Log Reduction	SD (log)	Evaluation
Mixed biofilm (*C. difficile*, *B. spizizenii*, *B. cereus*)	6.81 ± 0.12	<1.00 (LOD)	>6.8	0.28	Effective

**Table 6:** Test Group A: Sterilization with EC STER (4.5 g/L, contact time 1 min).

Strain Mix	Initial Count (log_10_ CFU/mL)	Final Count (log_10_ CFU/mL)	Log Reduction	SD (log)	Evaluation
Mixed biofilm (*C. difficile*, *B. spizizenii*, *B. cereus*)	6.81 ± 0.12	<1.00 (LOD)	>6.8	0.28	Effective

## Data Availability

The original contributions presented in the study are included in the article, further inquiries can be directed to the corresponding author.

## Abbreviations

The following abbreviations are used in this manuscript:

NCTC	National Collection of Type Cultures
ATCC	American Type Culture Collection
TSA	Tryptic Soy Agar
CFU	Colony-Forming Unit
BHIS	Brain Heart Infusion with Supplements
ICM	Initial Contamination Microbial load
